# Embryonic stem cells become mechanoresponsive upon exit from ground state of pluripotency

**DOI:** 10.1098/rsob.180203

**Published:** 2019-01-09

**Authors:** C. M. Verstreken, C. Labouesse, C. C. Agley, K. J. Chalut

**Affiliations:** 1 Cavendish Laboratory, Department of Physics, University of Cambridge, Cambridge CB3 0HE, UK; 2 Wellcome Trust/Medical Research Council Stem Cell Institute, University of Cambridge, Cambridge CB2 1QR, UK

**Keywords:** stem cells, mechanosensing, pluripotency, immediate early genes, mechanical signalling

## Abstract

Stem cell fate decisions are driven by a broad array of signals, both chemical and mechanical. Although much progress has been made in our understanding of the impact of chemical signals on cell fate choice, much less is known about the role and influence of mechanical signalling, particularly in embryonic stem (ES) cells. Many studies use substrates with different stiffness to study mechanical signalling, but changing substrate stiffness can induce secondary effects which are difficult to disentangle from the direct effects of forces/mechanical signals. To probe the direct impact of mechanical stress on cells, we developed an adaptable cell substrate stretcher to exert specific, reproducible forces on cells. Using this device to test the response of ES cells to tensile strain, we found that cells experienced a transient influx of calcium followed by an upregulation of the so-called immediate and early genes. On longer time scales, however, ES cells in ground state conditions were largely insensitive to mechanical stress. Nonetheless, as ES cells exited the ground state, their susceptibility to mechanical signals increased, resulting in broad transcriptional changes. Our findings suggest that exit from ground state of pluripotency is unaffected by mechanical signals, but that these signals could become important during the next stage of lineage specification. A better understanding of this process could improve our understanding of cell fate choice in early development and improve protocols for differentiation guided by mechanical cues.

## 1. Introduction

Stem cells receive and respond to a range of potentiating signals, both mechanical and chemical, from their local environment. Together, these signals create a balance to either maintain cells in a self-renewing state or push them towards differentiation [1–3]. The regulation of this balance by chemical signals has been widely studied; however, less is known about the role of mechanical signals, even though previous work has also indicated their potential relevance [4,5]. For example, matrix elasticity has been shown to influence lineage specification in adult stem cells [6–8].

On the other hand, little is known about how pluripotent stem cells sense and respond to direct mechanical signals. This is particularly true in conditions of naive pluripotency, or ground state of pluripotency, in which mouse embryonic stem (ES) cells closely resemble, both transcriptionally and morphologically, cells in the inner cell mass of the mouse embryo 4.5 days after fertilization [9]. Naive ES cells typically possess a rounded, tightly aggregated morphology; however, as they exit naive pluripotency, they exhibit increased spreading and the mechanical characteristics begin to change [10]. This change in morphology, along with actuation of mechanosensitive signalling pathways such as ERK [11,12], led us to hypothesize that ES cells become susceptible to mechanical signals only as they exit naive pluripotency and begin the process of differentiation.

A variety of techniques have been used to probe cells’ response to forces, ranging from pulling directly on integrins [13,14], or applying large-scale forces to cells [15,16], to plating cells on substrates with adjustable stiffness [17]. Multiple studies have shown the effects of plating cells on hydrogels with different stiffness to measure the impact of the mechanical microenvironment on cell phenotype [18–21]. However, this typically induces a wide range of effects in cells, including changes to cells' inclination for cell–cell versus cell substrate attachment, how cells adhere to the substrate, cell density and the level and nature of cell–cell signalling [22]. In this study, we have grown ES cells on elastic substrates and applied forces directly to the cells by stretching the substrate [23–25]. In this way, we exert forces directly on cells without inducing additional changes in the environment. This allows us to study cells’ direct response to mechanical stress, independent from other potential secondary effects that could also affect cell behaviour. As such, we argue that the downstream biological effects induced by stretching cells' substrate should be a direct result of the forces applied to the cells.

In this paper, we present a convenient, inexpensive and versatile set-up to exert mechanical stresses on cells, and introduce a novel functionalization protocol suitable for ES cells. Our set-up allows the investigation of the downstream biological effects of stretching in ES cells. The immediate response includes an increase in intracellular calcium and a transcriptional upregulation of the immediate and early genes (IEGs). On longer time scales, we found that the functional mechano-response was negligible in conditions of naive pluripotency, but that stretching had a potent effect as they exited naive pluripotency. Our results suggest that pluripotent cells become functionally mechanosensitive only upon exit from the naive state.

## 2. Material and methods

### 2.1. Cell culture

ES cells from 129/Sv strain mice, a kind gift from Jennifer Nichols's laboratory at the University of Cambridge, were cultured on gelatin-coated (Sigma) tissue culture plastic at 37°C and 7% CO_2_ and split every 3 days. Serum + LIF (SL) media consists of Glasgow Eagle's Minimal Essential Medium supplemented with 10% FBS, l-glutamine, non-essential amino acids, sodium pyruvate, β-mercaptoethanol and LIF. N2B27 consisted of neurobasal media, DMEM, l-glutamine, N2, B27 and β-mercaptoethanol. 2i media was N2B27 supplemented with CHIR99021 and PD0325901 [11]. Rex1GFPd2 cells were a kind gift from Austin Smith's laboratory at the University of Cambridge [26].

### 2.2. Stretcher set-up and PDMS membrane manufacture

The device was designed using Inventor 2016 (Autodesk) and printed on a Makergear M2 Rev E using PLA. An automated version of the stretcher included a high torque motor, an L293D motor driver chip (Texas Instruments) and a Raspberry Pi. Functionalized membranes of around 750 µm thick were manufactured by spincoating PDMS (1 : 20) on silicon wafers, then baking for 2.5 h at 75°C. With the edges covered, the membrane was exposed to oxygen plasma (2 min exposure at 100 W, Diener). They were then treated for 3 h with a solution consisting of 94% ethanol, 0.5% acetic acid and 0.1% Bind Silane (PlusOne, GE), followed by 1 mM BS^3^ (Thermo Fisher) and an overnight incubation with 100 µg ml^−1^ fibronectin (Corning) or laminin (Sigma).

### 2.3. Immunofluorescence stainings

Cells were fixed for 15 min in 4% PFA (Sigma), then blocked and permeabilized for 10 min in 0.05% goat serum (Sigma) and 0.3% Triton X-100 (Sigma), followed by incubation with 1 : 50 phospho-paxillin antibody (2541 Cell Signaling Technology) in 0.3% Triton X-100 and 0.002% BSA (Gibco) overnight at 4°C. Samples were then incubated with an Alexa Fluor-conjugated secondary antibody or 1 : 800 phalloidin (Abcam) for 90 min, followed by incubation with 2 µg ml^−1^ 33342 Hoechst for 10 min.

### 2.4. Calcium analysis and live imaging

Changes in intracellular calcium concentration were measured in ES cells plated in SL on fibronectin-coated membranes for 24 h. After 30 min staining using 10 nM X-Rhod-1 (Thermo Fisher), the media was replaced with SL with 10 mM HEPES (Sigma) at pH 7.4 containing 2 µM Hoechst 33342. 30 min later, cells were imaged on a Leica CTR7000 HS epifluorescence microscope using a 20×/0.4 n.a. objective. Images were first taken before stretching. Then the membrane was stretched, and the field of view and the focal plane were adjusted. This lasted approximately 1 min, and then images were taken of cells in a stretched state. Images were analysed using Volocity (PerkinElmer) and Fiji [27], with the X-Rhod-1 signal used to identify cells' calcium signal. To change the intracellular calcium concentration, cells were treated for 30 min with 30 µM BAPTA/AM (Insight Biotechnology). To measure nuclear shape and alignment, cells were incubated with Syto13 (ThermoFisher), at a 5 µM concentration for 30 min, then imaged immediately (control) or stretched and imaged 90 min later (stretched). An ellipse was fitted to the nucleus (Fiji) and the major and minor axes were extracted. To measure the degree of apoptosis during stretching, CellEvent caspase-3/7 green dye (R37111, Thermofisher) was added to the medium 30–60 min prior to stretching.

### 2.5. qPCR

For gene expression measurements, RNA was isolated using the RNAeasy mini kit (Qiagen) including DNAse treatment (Qiagen) and reverse transcribed using Superscript III (Invitrogen). TaqMan assays were used for the RT-qPCR reactions (Egr-1: Mm00656724, c-fos: Mm0487425, c-jun: Mm00495062, c-myc: Mm00487804, Nanog: Mm02384862, Klf4: Mm00516104, Esrrb: Mm0044241, Acta2: Mm00725412).

### 2.6. RNA sequencing and bioinformatics analysis

RNA-Seq libraries were created using ribosomal RNA depletion (Ribo-Zero rRNA Removal Kit MRZH11124, Illumina) and produced from rRNA-depleted RNA using the NEXTflex Directional RNA-Seq Kit V2 (Bioo Scientific) with 12 cycles of PCR amplification, then Illumina sequenced using single-end sequencing for the SL-type samples and using pair-end sequencing for the 2i-type samples. The sequencing data were analysed using DESeq2 [28] with genes considered as significantly regulated when *p*_adj_ < 0.1. Gene ontology analyses were performed using goseq with *p*_overrepr_ < 0.05 [29], GOrilla with *q* < 0.1 [30] and GSEA with *q* < 0.25 [31].

### 2.7. Western blots

Protein lysates were collected in RIPA buffer (Cell Signaling) with protease and phosphatase inhibitors (Sigma). After denaturation in SDS, samples were loaded in a gradient mini-protean gel 8–14% and transferred to a nitrocellulose membrane. After transfer, the membranes were blocked (BSA 5%, 2 h) before probing with anti phospho-Tyr118-paxillin (#2541, CellSignaling, 1 : 1000), anti phospho-ERK (#4370, CellSignaling, 1 : 1000) and anti-LaminB1 (ab16048, Abcam, 1 : 10000) overnight, at 4°C. Anti-rabbit-HRP secondary antibodies (1 h) were used before revealing on film with ECL Prime revealing agent (GE Healthcare).

## 3. Results

### 3.1. Development of a cell substrate stretcher

In order to investigate the influence of direct mechanical cues on ES cells, we developed a device to apply forces to cells attached to an elastic polydimethylsiloxane (PDMS) substrate. This approach allowed us to investigate the exclusive effect of tensile forces on short time scales without inducing changes to cell density or relative affinities of cells to other cells or to the substrate.

**Figure 1.**
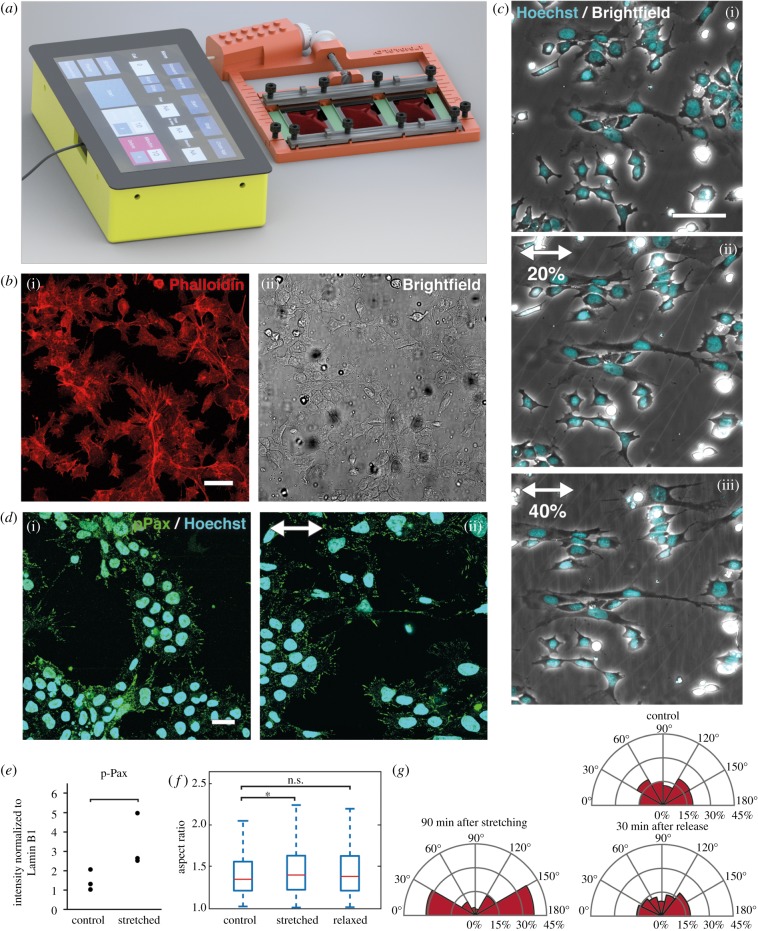
A cell substrate stretcher to apply direct forces to cells on an elastic substrate. (*a*) Automated stretcher, driven by a motor and used to stretch three membranes in parallel. The device is connected to a Raspberry Pi, which can be used to automate custom stretching protocols. (*b*) ES cells in SL media conditions on PDMS membranes functionalized with fibronectin, with phalloidin marking actin fibres (i) and bright-field (ii). Scale bar 50 µm. (*c*) Cells undergoing stretching: bright-field and nuclei (cyan) in (i) unstretched conditions and after stretching (ii) 20% and (iii) 40%. Images were taken within 5 min after stretching. Scale bar 25 µm. (*d*) Cells fixed and stained for phosphopaxillin (green) and nuclei (cyan) before (i) and 2 min after (ii) exposing cells to 40% stretch. The direction of stretching is indicated using arrows. Scale bar 25 mm. (*e*) Quantification of western blots for phospho-paxillin (p-Pax) in cells grown in unstretched control conditions, and 2 h after stretching. Intensity was normalized to Lamin B1 intensity (see electronic supplementary material, figure S2B–C). (*f*) Aspect ratio (ratio of major to minor axis of an elliptical fit) of nuclei stained with Syto13 in unstretched control conditions, 90 min after stretching cells 35%, and 30 min after subsequently releasing the stretch. (*) indicates significant differences (*p* < 0.05, *n* > 300 cells). (*g*) Axis of alignment of nuclei in unstretched control conditions, after stretching and after releasing the stretch. Conditions are identical to (*f*). Percentage indicates proportion of cells with nuclei in a given orientation with respect to the direction of stretch.

The device we present here has been designed using CAD software and can be printed from fully biocompatible plastic on most basic 3D printers. As such, our set-up is distinguished by a combination of experimental convenience and biological precision [24,32,33]. Multiple versions were optimized for specific purposes such as live cell imaging, immunofluorescence stainings or molecular biology assays. One variant (figure 1*a*) incorporates an actuator and supports three replicate membranes. A stretching protocol can then be entered on a graphical user interface (GUI) on a Raspberry Pi. The 3D models have been made publicly available (see electronic supplementary material) in order to allow others to adapt the platform for their own applications.

In all experiments presented here, cells were seeded on a membrane with sub-millimetre thickness, manufactured from PDMS. We found that the level of cell attachment produced by conventional functionalization techniques such as sulfo-SANPAH was insufficient for ES cells which are not highly adhesive compared with many cells. Therefore, we developed a novel functionalization protocol using an amine-to-amine cross-linker (electronic supplementary material, figure S1), resulting in a coating that enables binding of different possible extracellular matrix (ECM) proteins such as fibronectin, laminin or collagen. While the central area of the membrane was functionalized, the boundaries remained hydrophobic such that culture media remained on the membrane without requiring a media well that might interfere with stretching. We observed that ES cells in serum + LIF (SL) media attached well to the PDMS membrane coated with either laminin or fibronectin, and that the morphology and cytoskeleton of ES cells were similar to cells on tissue culture plastic (figure 1*b*). We used the fibronectin coating for SL culture because it facilitates spreading of ES cells as opposed to aggregation [34].

We first tested the capabilities of our device by stretching ES cells in SL conditions, a media condition that allows for a high degree of heterogeneity in ES cell phenotype. Cells remained well attached to the membrane after stretching, as demonstrated by their morphology (figure 1*c*), with 98 ± 2% of cells remaining attached 90 min after stretching (*n* = 300 cells across 3 membranes). For a stretch of 20% and 40% the extremities of the cells extended, respectively, 19 ± 4% and 38 ± 5% (*n* = 15) in the direction parallel to the macroscopic stretch, and retracted, respectively, 11 ± 4% and 22 ± 4% in the direction perpendicular to the stretch, showing that cells’ strains were proportional to the global stretch. Moreover, foci of Tyr397-phosphorylated paxillin (p-Pax), a marker of substrate-attached focal adhesions, were well defined in both unstretched samples and in samples with 35% stretch (figure 1*d*), with a twofold increase in the amount of phospho-paxillin, as quantified by western blot (figure 1*e*; electronic supplementary material, figure S2B). To test whether cells experienced continual mechanical stress as a result from the stretch even after extended periods of time, we measured the shape and alignment of the nucleus. The aspect ratio of the nuclei, measured as the ratio of the major over the minor axis of an elliptical fit, was increased significantly 90 min after stretching (*n* > 300, *p* < 0.05) (figure 1*f*). In addition, similar to other cell types, nuclei also became significantly more aligned along the direction of stretch (figure 1*g*) [35]. When subsequently releasing the stretch, both the aspect ratio and the alignment partially reverted to the unstretched state 30 min after stretching (figure 1*g*). Taken together, these results demonstrate that cells are well-adhered when plated on the functionalized PDMS membranes, responded fully to the applied stretch and experienced continued external mechanical stress after stretching.

### 3.2. Changes to calcium in response to stretching

**Figure 2.**
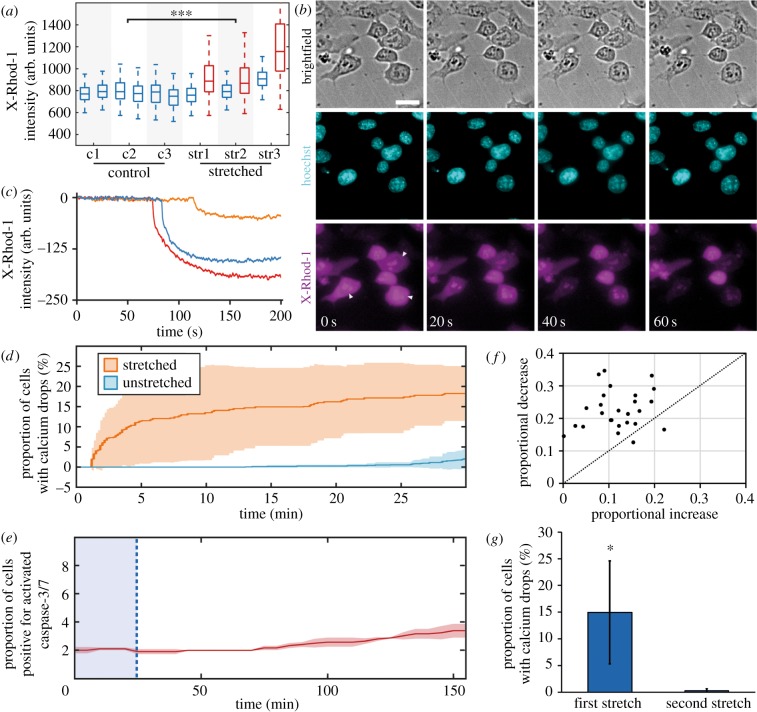
Changes to intracellular calcium concentrations following stretching. (*a*) The change in the mean X-Rhod-1 intensity of cells between two subsequent images (taken 3 min apart). Three membranes were left unstretched (c1–3), and three membranes were stretched 35% (str1–3). Boxplots correspond to 400 cells per experiment on unstretched (blue) and stretched (red) samples. An *n*-way ANOVA showed that the explanatory variables ‘strain’ (before versus after stretching) and ‘sample type’ (control c1–c3 versus stretched str1–3) were both statistically significant. (*b*) Intensity of X-Rhod-1 (magenta) during a 60 s interval approximately 4.5 min after stretching, with rapid X-Rhod-1 decrease in three cells indicated using arrows. Nuclei shown in cyan. Scale bar 25 µm. (*c*) Representative drops of the X-Rhod-1 intensity in three cells during a 200 s interval. (*d*) Proportion of cells exhibiting calcium drops during 30 min after stretching (orange) or in unstretched conditions (blue) in a population of around 400 cells each on three membranes. Shaded area corresponds to standard deviation of three membranes. (*e*) Proportion of cells with positive marker for activated caspase-3/7, during 30 min before (blue shaded area) and 130 min after stretching. Shaded area corresponds to the standard deviation of four membranes. (*f*) Magnitude of the proportional increase in X-Rhod-1 during stretching, compared to the subsequent decrease in X-Rhod-1 during a calcium drop (*n* = 25 cells). (*g*) Proportion of cells exhibiting calcium drops during the first 15 min after initial stretch, and during 15 min after a second stretch, preceded by a 90 min initial stretch, a 30 min relaxation. (***) indicates significant differences (*p* < 0.001).

Calcium has been described to be involved in the mechanosensitive response in multiple cell types, either directly through stretch-activated calcium channels or through integrins [36,37]. In order to show that ES cells respond to mechanical signals beyond changes in morphology, we first examined calcium signalling upon mechanical stretch in cells grown in SL conditions. We used live imaging of the fluorescent marker X-Rhod-1 to measure intracellular calcium levels during cell stretching. It is important to note that, due to slight shift and thinning of the membrane upon stretch, a refocusing of the cells after stretch was necessary, which required approx. 30–60 s. Therefore, the calcium signal represents intracellular calcium shortly, but not immediately, after stretch. Nonetheless, by imaging X-Rhod-1, we observed a significant increase in the intracellular calcium after stretching (figure 2*a*). This number is probably underestimated given the lag time between stretch and imaging.

After this initial increase in intracellular calcium immediately following the stretch, some cells exhibited a corresponding sudden drop in calcium concentration within minutes after stretching (figure 2*b*). In these cells, the intracellular calcium concentration decreased rapidly within a time span of 1–2 min (figure 2*c*). Within the first 30 min, around 18% of cells in the stretched samples exhibited a calcium drop, compared to only 4% in the unstretched samples (figure 2*d*). The number of drops we observed in the stretched samples is also likely to be underestimated compared with the actual number of drops due to the lag time between stretching and imaging. The presence of these calcium drops was uncorrelated to cell shape, size or location. We also showed that they are not a signature of apoptosis induced by stretching, as only 2% of cells were marked positive for active caspase-3/7 30 min after stretching (figure 2*e*). The mean of the magnitude of cells' calcium drops was proportionally larger but of a similar magnitude as cells’ mean increase in the original calcium level during stretching (figure 2*f*), indicating that the calcium drops are directly connected to the increase upon stretch. Since imaging the drops is technically simpler than imaging the initial increase, we used the presence of these drops as a proxy for the existence of calcium flux as a response to stretch. Taken together, our findings suggest that the changes in intracellular calcium indicate a cellular mechanical response.

Next we exposed cells to a cycle of repeated stretching, consisting of an initial stretch of 35% maintained for 90 min, then relaxing the membrane for 30 min and then repeating the stretch. No cells exhibited signs of a calcium flux after exposing the cells to a second stretch (figure 2*g*), from which we infer that the calcium level of the cells is insensitive to further mechanical signals for at least 100 min after the first mechanical ‘trigger’ induced by stretching. Therefore, our data suggest that cells undergo a mechanical equilibration after the first stretch that renders them less mechanically sensitive to further stretches. We next asked whether the changes at the level of calcium signalling are associated with changes at the transcriptional level.

### 3.3. Transcriptional changes caused by stretching in ES cells

Earlier studies indicated that the intracellular concentration of calcium can affect the expression of the IEGs [38,39]. As a part of cells' frontline transcriptional response, this gene family is typically induced rapidly on time scales between 15 min and 2 h [40,41], and can be re-activated every 2 h [42].

**Figure 3.**
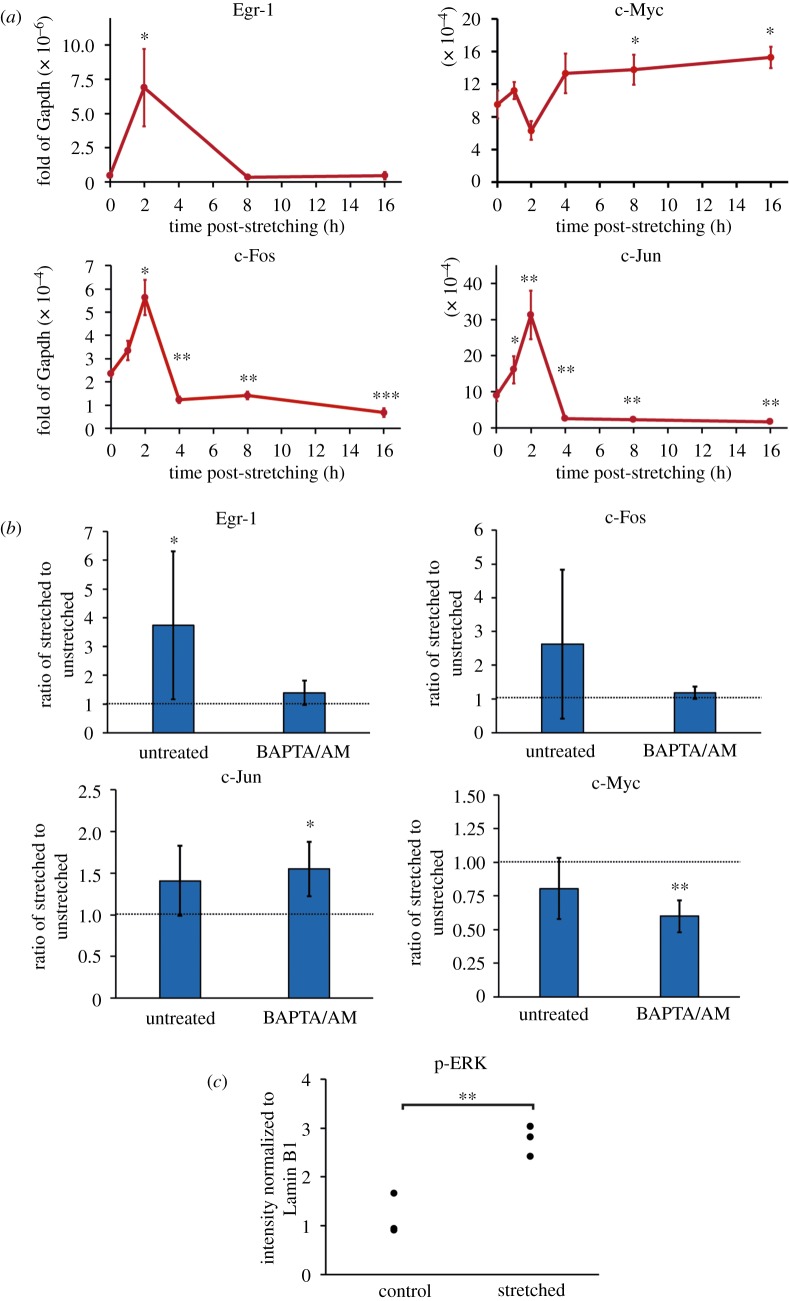
Stretching induces calcium-dependent changes to IEG transcription. (*a*) Expression levels of 4 IEGs after single stretch of 35% in naive conditions. ES cells in SL were exposed to a single stretch of 35% at *t* = 0 h, which was maintained during the next 16 h. The 0 h timepoint corresponds to the control conditions. (*b*) Ratio of the IEG expression level in stretched to unstretched cells in SL medium. Cells were stretched 35% for 2 h in untreated conditions or treated with the calcium chelator BAPTA/AM. (*c*) Quantification of phospho-ERK by western blot (see electronic supplementary material, figure S2C) in control condition and in cells exposed to 2 h of 25% single stretch in SL. (*), (**) and (***) indicate significant differences at *p* < 0.05, *p* < 0.01 and *p* < 0.001, respectively.

Given that we found a mechanosensitive calcium response, we hypothesized that we would see a similar increase in IEG transcription in response to stretching. Therefore, we first measured the expression of known IEGs Egr-1, c-jun, c-fos and c-myc after exposing ES cells in SL to a single stretch of 35%. We found that stretching induced a significant change in the transcription of all four IEGs we measured, with Egr-1, c-jun and c-fos reaching a peak 2 h after stretching (figure 3*a*). In contrast to the other IEGs, c-myc exhibited a long-term upregulation in response to stretching. Given previous results that IEGs could be re-activated every 2 h [42], we also added a frequency-dependent component by releasing and then re-applying the stretch after 1 and 2 h, respectively. This frequency modulation did not have an impact on the expression of the IEGs (electronic supplementary material, figure S2A), indicating that the mechanism leading to the activation of the IEGs was surprisingly insensitive to further mechanical signals.

Based on the observation that intracellular calcium and IEG transcription were both insensitive to further mechanical signals, we next investigated whether the transcriptional dynamics of the IEGs were driven by changes in the calcium concentration in response to stretching. To this end, we treated cells with a calcium chelator, BAPTA/AM, which binds calcium ions and thereby reduces the amount of free intracellular calcium in the cells [43]. As a result of this treatment, the increase in Egr-1 and c-Fos after stretching was eliminated (figure 3*b*). However, the BAPTA/AM treatment did not completely abrogate the stretch response of c-Jun and c-Myc, suggesting that the mechanism underlying transcriptional regulation of these two genes during stretch could differ from that of Egr-1 and c-Fos. The transcription of the IEGs is also regulated by ERK activity, and we indeed also observed a significant (*p* < 0.01) increase in the amount of phospho-ERK (p-ERK) as quantified by western blot (figure 3*c*; electronic supplementary material, figure S2C). These results demonstrate an unsurprising role for both calcium and ERK, which is known to be regulated by calcium, in the transcriptional activation of IEGs [44–46]. However, it also suggests that there may be a means through which the IEGs are stretch-activated that is either independent of or only partially dependent on calcium, such as regulation through focal adhesions [47], as supported by the increased p-Pax signalling on the stretched samples (figure 1*e*).

**Figure 4.**
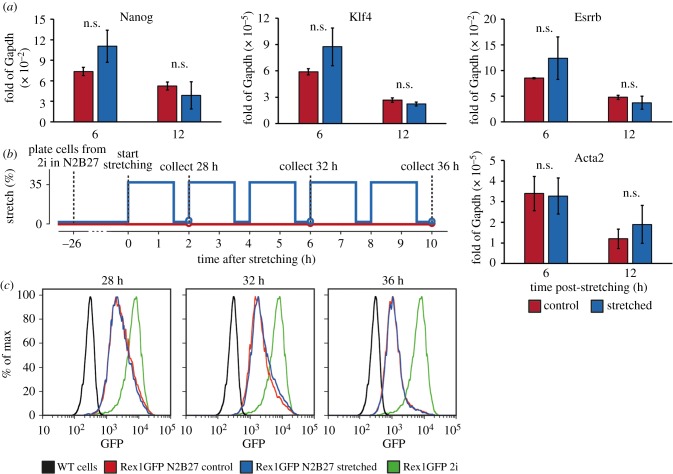
Stretching does not induce transcriptional changes in the naive pluripotency network. (*a*) Gene expression of pluripotency genes during differentiation. ES cells were plated from 2i into N2B27 at *t* = −12 h, and were exposed to a single continuous stretch of 35% at *t* = 0 h. n.s. indicates no significant differences. (*b*) Experimental protocol of Rex1::GFPd2 differentiation assay, with stretched (blue) and control (red) samples. (*c*) GFP flow distribution of Rex1::GFPd2 during differentiation in N2B27, 28 h, 32 h, 36 h after initiation of differentiation by replacing 2i with N2B27 (corresponding to 2 h, 6 h and 10 h after stretching). The positive (Rex1GFP 2i, green) and negative (WT cells, black) controls shown in each graph correspond to the respective profiles at 28 h only.

Considering that ERK plays an integral role in exit from naive pluripotency on the one hand [48], and on the other hand is highly involved in the response to external (mechanical) signals, we hypothesized that external mechanical stresses would affect exit from naive pluripotency in ES cells [12,49]. Notably, however, ERK-triggered transcription of IEGs and of downstream targets has been reported to depend on the frequency and duration of the ERK pulses [42,50]. As such, the increase in p-ERK and the IEGs would not necessarily lead to long-term downstream transcriptional effects. Exit from naive pluripotency in ES cells is typically triggered by removing the chemical differentiation inhibitors PD03, an inhibitor of MEK/ERK activity, and Chiron, and inhibitor of GSK3β activity, included in 2i media [26]. The initiation of differentiation begins with an exit from naive pluripotency which is typified by a decrease in naive pluripotency markers (such as Nanog, Klf4 and Esrrb). Within 24–36 h after removal of 2i media, cells have entirely exited the naive state and have become sensitive to lineage inductive cues [26]. To test the effect of stretching on exit from naive pluripotency, we plated ES cells from 2i into N2B27 on the stretcher, then stretched them 35% and performed qRT-PCR of cells collected 6 h and 12 h after stretching. We found that stretching did not alter the expression of the pluripotency genes Nanog, Klf4 or Essrb at either time point (figure 4*a*). In order to probe the robustness of this result, we performed this experiment across different media conditions and different cyclic stretch conditions (low and high frequency), finding no consistent changes in naive pluripotency markers across all conditions (electronic supplementary material, figure S3).

Since stretching induced negligible differences in the expression of the naive pluripotency genes we assayed, we employed a functional assay to explore the effects of stretching on naive pluripotency. We used Rex1::GFPd2 ES cells, in which the naive pluripotency marker Rex1 is attached to an unstable form of GFP, as presented in [48], with a half-life of approximately 2 h. The fluorescence intensity of this protein has been shown to correlate highly with the state of the naive pluripotency network [26], with Rex1::GFPd2 observable only in bona fide naive ES cells. We plated the Rex1::GFPd2 cells from 2i into N2B27 on the cell stretcher. To maximize the downstream effects on the naive pluripotency network, we then exposed cells to a repeated cycle of stretching and relaxation, starting 24 h after plating (figure 4*b*). Using flow cytometry at 28 h, 32 h and 36 h after the initiation of differentiation (corresponding to 2 h, 6 h and 10 h after stretching), we found that there was no difference in the expression of Rex1::GFPd2 (figure 4*c*). Therefore, our data indicate that, although stretching induces an upregulation of the IEGs, it surprisingly has no observable, long-term downstream functional impact on the exit from naive pluripotency.

### 3.4. Overall transcriptional changes in response to stretching

**Figure 5.**
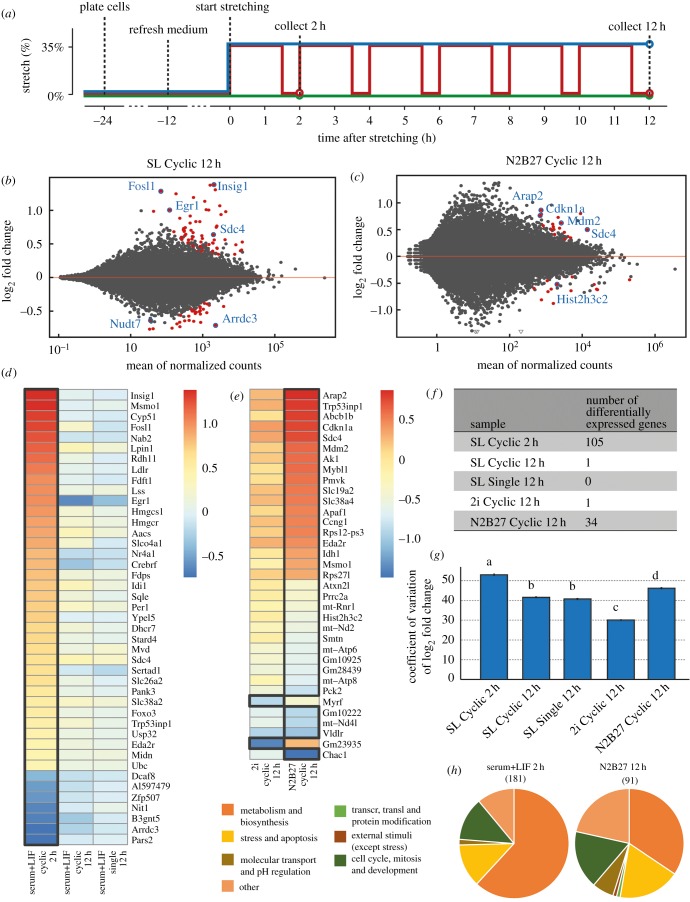
Stretching induces changes to the global transcriptome. (*a*) Protocol of stretching experiment used to collect cells used for RNA sequencing of cells in SL medium for samples exposed to cyclic stretching (blue), single stretch (red) and control (green). (*b,c*) Relationship between log_2_ fold change and normalized counts for (*b*) SL 36 h Cyclic 2 h and (*c*) N2B27 36 h Cyclic 12 h. Significantly regulated genes shown in red. (*d–e*) Ranked log2 fold changes of stretched to control conditions, for individual genes with adjusted *p*-value below (*d*) 0.01 and (*e*) 0.1 in (*d*) SL and (*e*) 2i and N2B27. (*f*) Number of differentially expressed genes for each condition. (*g*) Coefficient of variation of randomly selected sets of 1000 genes. Error bars correspond to SEM from 10 000 repeats. The letters on top of the bars indicate statistically significant differences as determined by Student's *t*-test. (*h*) Distribution of genes identified using the goseq package in seven broad types, according to their biological role, SL 36 h Cyclic 2 h and in N2B27 Cyclic 12 h.

In order to confirm that naive ES cells are relatively insensitive to mechanical signals and whether cells’ sensitivity to mechanical signals changes during the exit from naive pluripotency, we conducted RNA-Seq experiments of ES cells in stretching conditions in several media conditions. These media conditions included naive pluripotency conditions SL and 2i, and exit from naive pluripotency (i.e. differentiating) conditions in N2B27 alone. In order to maximize the chance that cells in the N2B27 condition had started to exit naive pluripotency and started differentiating, we initiated the stretching procedure 24 h after plating in N2B27. We exposed these cells to one of three procedures (figure 5*a*). Either cells underwent a stretching protocol with a repeated cycle consisting of 35% stretch for 90 min, then relaxation for 30 min (Cyclic), or cells were exposed to a single stretch of 35% (Single), or left unstretched (Control). We then collected cells after 2 h or after 12 h. We chose the timepoint as 12 h because, after that, the cells start to become more confluent, which would make any results difficult to interpret. For each condition, we compared gene expression of the stretched to the unstretched conditions, resulting in nine conditions (five stretched and four control conditions), allowing us to investigate the effects of media condition (SL, 2i), stretch programme (cyclic, single and control), stretch duration (2 h and 12 h) and exit from naive pluripotency (SL and 2i versus N2B27).

Comparing the log_2_ fold change of the stretched to the control samples, multiple genes were regulated in just two conditions: cyclic stretch in SL for 2 h (SL 36 h Cyclic 2 h) or in exit from naive pluripotency conditions (N2B27 36 h Cyclic 12 h) (figure 5*b*). The effect of stretching on the transcriptome was largest in SL at 2 h (figure 5*c*), with, for example, the IEGs Egr-1 and c-fos being significantly upregulated (figure 5*d*), as confirmed earlier by qRT-PCR (figure 3). By 12 h after stretching, the functional impact of stretching on ES cells in SL and 2i was non-existent. This confirms that, apart from initial mechanosensitive responses, stretching induces few transcriptional changes on longer time scales in ES cells. It further suggests that cells in naive pluripotency conditions are buffered in some way from mechanical signals. After exiting naive pluripotency, by contrast, the effects of stretching on pluripotent (but not naive) cells are much more persistent, with broad transcriptional changes 12 h after stretching (figure 5*e,f*).

We further studied the effect of culture conditions and cell state by analysing the heterogeneity of the transcriptional response. We chose sets of 1000 genes at random and calculated the coefficient of variation (defined as variance divided by the mean) of the change from control to stretched samples. Repeating this process 10 000 times, we found that the SL 2 h response contained the largest variability (figure 5*g*). The heterogeneity decreased 10 h later in SL and was not affected by the difference between single and cyclic stretch. The naive pluripotent condition of 2i exhibited the highest homogeneity, consistent with it being a highly transcriptionally constrained culture condition [51]. By contrast, the mechanosensitive response to stretching of differentiating cells in N2B27 was highly heterogeneous compared to other conditions probed at 12 h after stretching. Our data therefore suggest that, as they exit the naive state, pluripotent cells undergo a transition that renders them susceptible to mechanical signals and broadens the transcriptional response to those signals.

To identify the specific transcriptional responses, we performed a gene ontology analysis of the changes in the different conditions. We used the gene ontology package goseq [29] to identify the pathways that were enriched in response to stretching in the conditions SL 36 h Cyclic 2 h and N2B27 36 h Cyclic 12 h and found 181 and 91 pathways were upregulated, respectively. We then sorted the pathways in seven broad categories according to the biological function (figure 5*h*). In both conditions, pathways related to metabolism and biosynthesis were enriched, particularly those involving lipids and membrane components. Other responses to stretching included pathways centred on stress and apoptosis (including p53 and overall regulation of apoptosis), and cell cycle, mitosis and development (including cell proliferation, blood vessel development). Compared with the 2 h SL samples, the stress and cell cycle-related pathways were more prominent in differentiating cells in N2B27 after 12 h of stretching.

We then carried out the overrepresentation of pathways in all conditions using ranked GOrilla analysis [30] and using gene set enrichment analysis (GSEA) [31]. Multiple pathways were identified in all conditions using both methods, except in 2i, where GSEA only identified a single underrepresented pathway. While the stretch response involved a broad range of processes across all conditions (electronic supplementary material, figure S4), pathways related to metabolism were always affected, with secondary effects including changes to transcription and translation. Therefore, our results indicate that cells across conditions share a core response that involves stress and metabolism pathways.

## 4. Discussion

We present here a method to apply direct mechanical stresses to ES cells attached to a functionalized PDMS membrane. Compared with previous methods, a key innovation in our approach is its easy manufacturing and adaptation for specific applications, while simultaneously increasing biological power through automation and the inclusion of multiple replicates. We demonstrated that cells experience a strain proportional to the macroscopic stretch, reorient along the axis of stretching and experience an increase in p-Pax, a component of mature integrin-based adhesions. Stretching induced an increase in the concentration of intracellular calcium immediately after stretching, possibly through the involvement of the known stretch-activated calcium channels [39,52]. This was followed by an upregulation of a subset of the IEGs, such as Egr-1 and c-Fos, in a calcium-dependent manner.

Nevertheless, the intracellular calcium concentration was unaffected by subsequent stretches. Considering the role of stretch-activated calcium channels in regulating intracellular calcium concentration, this desensitization could be caused by adaptation of cellular properties, for example, membrane tension, to mechanical stress. Indeed, the strain on the membrane induced by stretching can lead to exocytosis, which would lead to a decrease in membrane tension and lower responsiveness of stretch-activated channels [53]. Moreover, if there is a difference in the regulation of membrane turnover or tension between naive ES cells and differentiating cells, as differences in morphology would suggest, this difference could affect sensitivity to dynamic mechanical stresses. Subsequent stretches also did not affect IEGs transcription (electronic supplementary material, figure S2). This is in contrast to the findings in [42], where continuous RAS activation of IEGs through ERK was demonstrated using a chemical signal with a frequency of 2 h in NIH3T3 cells, which influenced our choice of stretch frequencies. Future studies should aim to explain the discrepancy in frequency dependence between mechanical and chemical actuation of IEGs. There are several aspects of the discrepancy worth considering: first, the buffering of frequency-dependent mechanical signals could be specific to ES cells; second, the IEGs could exhibit a frequency-dependent response to mechanical stress, but on a different time scale than the one we applied; finally, the lack of observed frequency-dependent stretch-activated response of IEGs may underlie a key difference between how mechanical signals and chemical signals are transduced.

The results in this study identify the concrete effects of mechanical signals on pluripotent cells and how these signals depend on the broader cell state. In past studies, cell spreading (i.e. adhesion and ensuing cytoskeletal tension), has been designated a ‘master regulator’ of the mechanical sensitivity in cells [54,55]. Therefore, studies observing differences in pluripotency potential in ES cells cultured on soft and stiff substrates [19,56] could be primarily reflecting the baseline adhesion of the cells to their substrate [22,57]. Furthermore, cells' initial spreading likely has downstream effects on cell–cell attachment and local density. In our study, we control for this effect by ensuring that cells are well-adhered and spread on the substrate before the experiment. In this manner, we are able to probe the effect of direct, extrinsic mechanical stresses on ES cells. Importantly, studies of substrate stiffness-dependent differences in ES cell function may reflect differences in heterogeneity in the sample, and not effects of mechanical cues on naive ES cells. In other words, the soft substrates may be less supportive of ES cells that are prone to differentiation.

When we applied direct tension to the cells, we found that cells quickly respond with broad transcriptional changes particularly reflecting metabolic and biosynthesis pathways (often related to membrane components). However, we found that naive pluripotent ES cells, unlike other cell systems studied in this way [58–60], are largely insensitive to mechanical signals on the longer term. Importantly, only as ES cells exit naive pluripotency do they become sensitive to these external mechanical inputs, affecting stress and metabolism pathways in particular. As such, we conclude that naive pluripotency can act as another regulator of mechanical sensitivity, controlling the effect of mechanical signals on the transcriptome. Considering the minimal transcriptional response to mechanical stretch, our transcriptome-wide data do not provide insight into the buffering mechanism. This suggests that the buffer to mechanical signals is achieved upstream of transcription, probably through direct post-translational protein modifications. These protein-level effects of stretching should be investigated in future research so as to better contextualize the mechanical buffering we observed in ES cells. Since recent work suggests that naive pluripotency guards ES cells against lineage inductive signals [61], it is possible that resistance against mechanical signals is also an intrinsic aspect of the regulation of the maintenance of pluripotency, and mechanical signals become an important aspect of differentiation. It will be very valuable, in the future, to focus investigations on how mechanical cues influence lineage specification.

## Supplementary Material

Supplementary Figures

## Supplementary Material

Stretcher3Dmodel

## Data accessibility

The accession number of the deposited RNA sequencing data is S-BSST217 on BioStudies (www.ebi.ac.uk/biostudies/studies/S-BSST217).
