# Supplementary material for: Embryonic stem cells become mechanoresponsive upon exit from ground state of pluripotency
Source: Open Biol. 2019 Jan 9;9(1):180203. doi: 10.1098/rsob.180203 (PMC6367133; doi:10.1098/rsob.180203)
Supplement: Supplementary Figures [file rsob180203supp1.docx]

## **SUPPLEMENTARY INFORMATION**


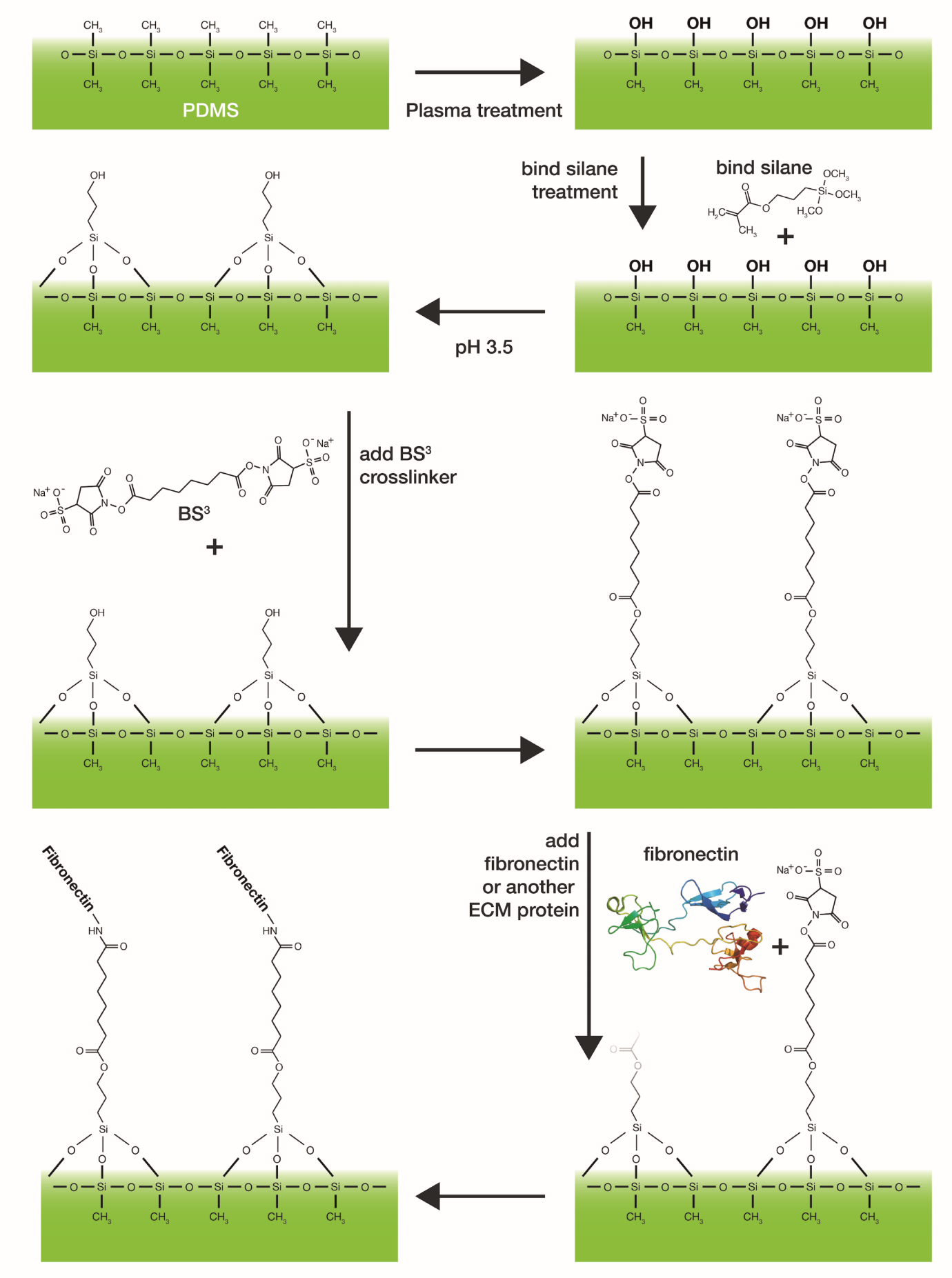


**Figure S1: functionalisation of PDMS surface with ECM proteins**

Functionalisation of PDMS membranes. First plasma treatment exposes silanol groups on the surface of the PDMS membrane. Then, bind silane molecules bind to up to 3 oxygen molecules on PDMS surface. Third, one end of the BS3 crosslinker is connected to the groups on the surface of the PDMS. After washes, adding fibronectin or alternative ECM proteins bind to the other end of the crosslinker.


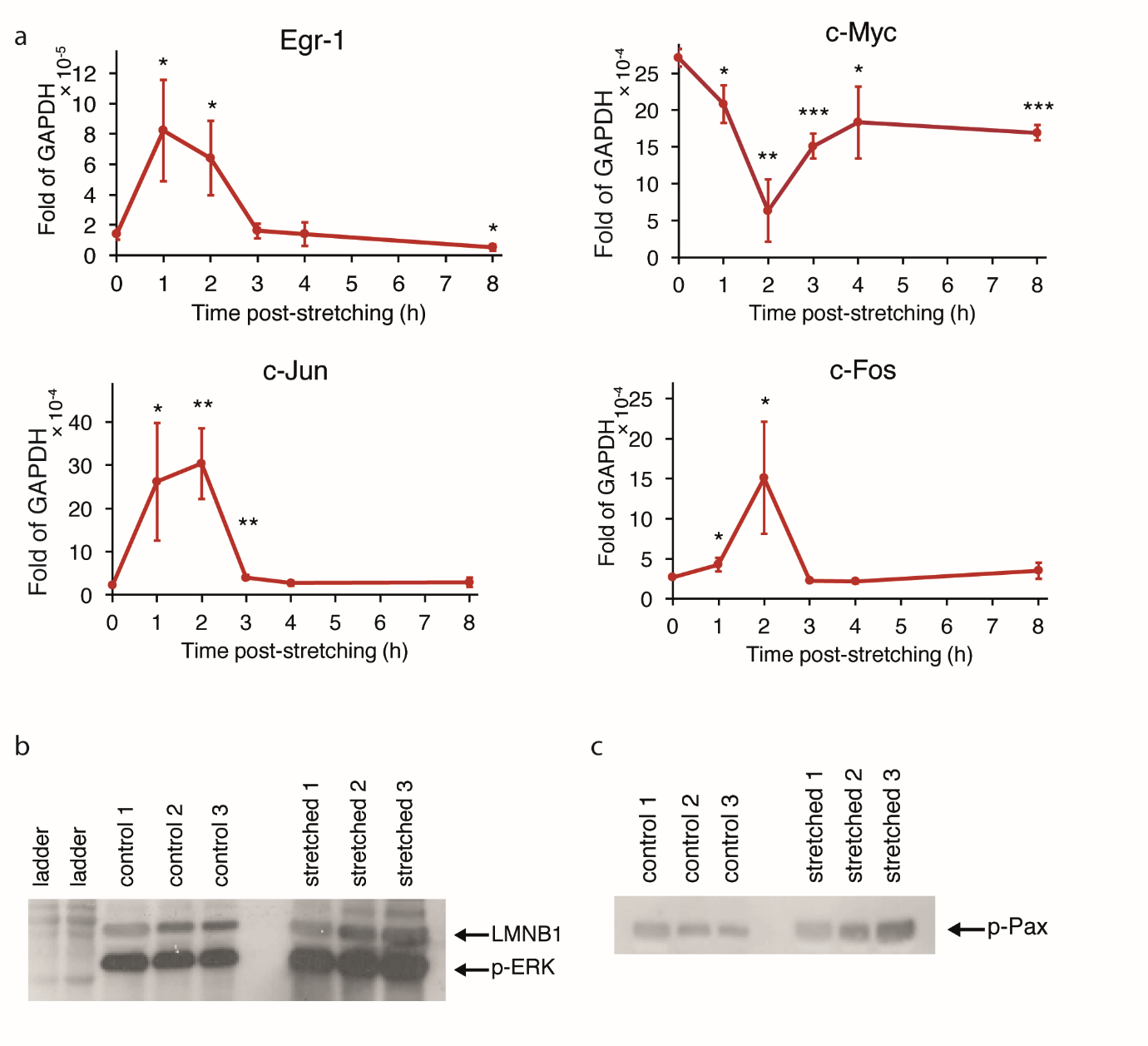


**Figure S2: Changes in transcription and protein concentration in response to stretching**

(a) Expression levels of the IEGs Egr-1, c-Myc, c-Jun, c-Fos after a protocol consisting of stretching 35% at t = 0 h, then relaxing at t = 1 h, and stretching again permanently at t = 2 h. The 0 h timepoint corresponds to the control conditions. Cells were exposed to identical conditions at the 0 h and 1 h timepoint as at the same timepoints in the experiment shown in Fig 3a. (*), (**) and (***) indicate significant differences at p < 0.05, p < 0.01 and p < 0.001 respectively. (b-c) Western blot of (b) p-ERK and Lmnb1, and (c) p-Pax.


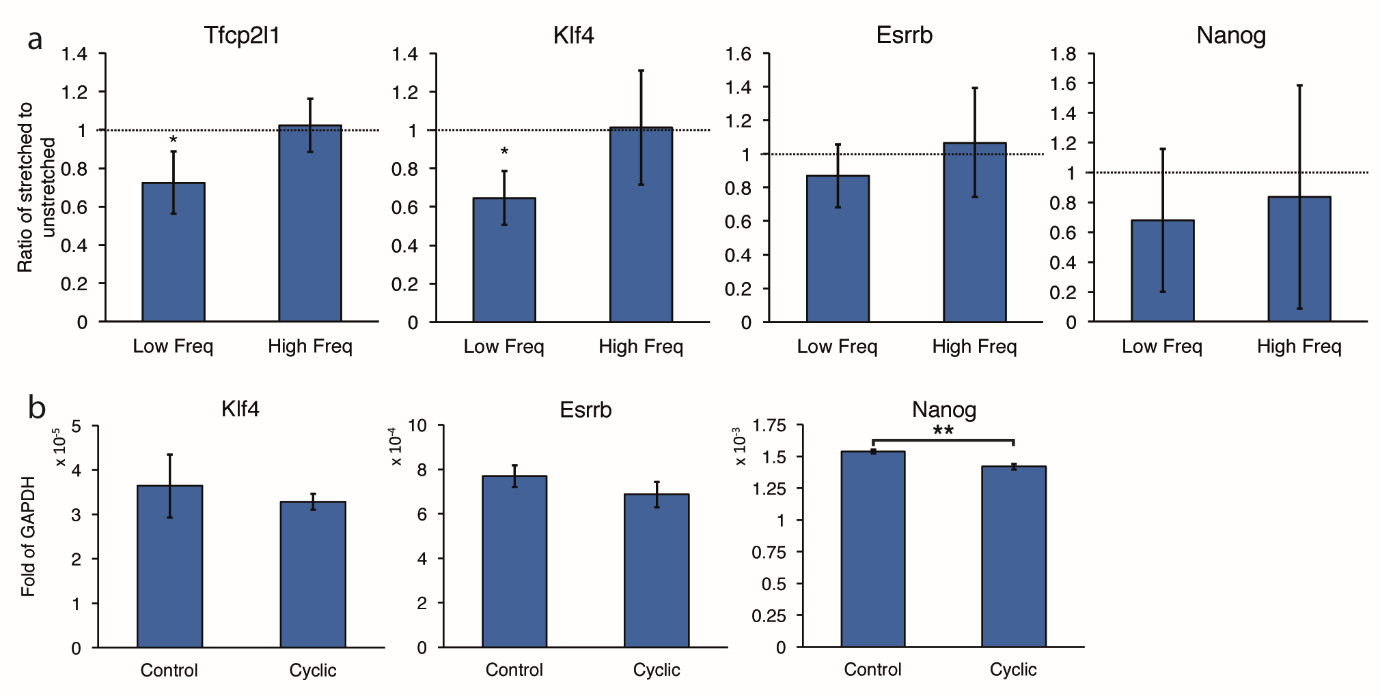


**Figure S3: Transcription of the pluripotency genes in response to stretching**

(a) Ratio of expression in stretched to unstretched conditions, for four pluripotency genes in SL media conditions. Cells underwent a cyclic stretching programme consisting either of stretching 35% for 90 min, then relaxation for 30 min (Low Freq) or stretching and relaxing at a frequency of 0.01 Hz (High Freq). (b) Transcription of Klf4, Esrrb and Nanog in response to stretching during exit from pluripotency. Cells were plated in 2i, and 12 h later, the media was changed to N2B27. Then, 12 h later, they were exposed to a cyclic stretching protocol consisting of a repeated cycle of 90 min of 35% stretch, then 30 min relaxation. Samples were collected 12 h after stretching. (*), (**) indicate significant differences at p < 0.05 and p < 0.01 respectively, N.S. indicates no significant difference.


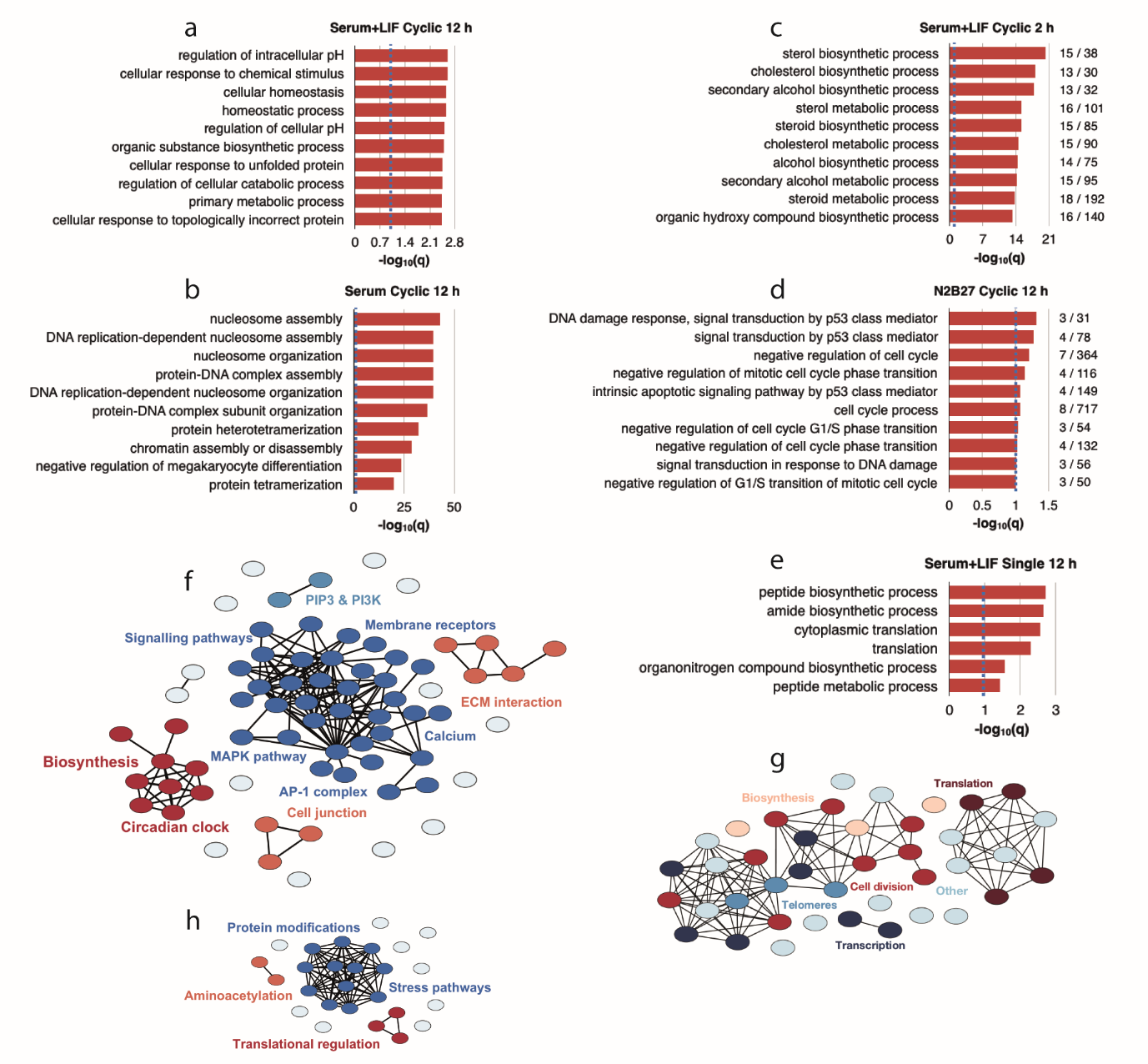


**Figure S4: Enriched pathways after stretching**

(a-d) Ten most highly enriched pathways (or less if fewer pathways are significantly enriched with q < 0.1) using (a-b) GOrilla of significant genes, with the proportion of genes per pathway that are significantly enriched indicated on the right and (c-d) GOrilla of the gene lists ranked by p-value. The q = 0.10 limit is indicated using a blue dashed line. (e-f) Interconnectivity map of pathways enriched in (e) SL 36 h Single 12 h (f) SL 36 h Cyclic 2 h, with clusters labelled according to the general biological function. Connections between nodes indicate that pathways have genes in common. Map produced using GSEA package and Cytoscape.
